# Genome Sequence of *Ustilaginoidea virens* IPU010, a Rice Pathogenic Fungus Causing False Smut

**DOI:** 10.1128/genomeA.00306-16

**Published:** 2016-05-05

**Authors:** Toshitaka Kumagai, Tomoko Ishii, Goro Terai, Myco Umemura, Masayuki Machida, Kiyoshi Asai

**Affiliations:** a Fermlab Inc., Koto-ku, Tokyo, Japan; b Technology Research Association of Highly Efficient Gene Design, Sapporo, Hokkaido, Japan; c INTEC Inc., Koto-ku, Tokyo, Japan; d Bioproduction Research Institute, National Institute of Advanced Industrial Science and Technology (AIST), Sapporo, Hokkaido, Japan; e Department of Computational Biology and Medical Sciences, Graduate School of Frontier Sciences, University of Tokyo, Kashiwa, Chiba, Japan

## Abstract

*Ustilaginoidea virens* is a rice pathogenic fungus that causes false smut disease, a disease that seriously damages the yield and quality of the grain. Analysis of the *U. virens* IPU010 33.6-Mb genome sequence will aid in the understanding of the pathogenicity of the strain, particularly in regard to effector proteins and secondary metabolic genes.

## GENOME ANNOUNCEMENT

*Ustilaginoidea virens* is a plant pathogenic fungus that causes false smut disease in rice. The disease is widespread in the major rice-growing areas of Asia, including Japan, and seriously damages both the yield and quality of the grain (1, 2). The fungus is considered as a biotrophic pathogen (3) that obtains nutrition from living host cells and secretes effector proteins to suppress or regulate the immune response of plants to invasion (4). In this report, the genome sequence of the *U. virens* strain IPU010 was determined. It was isolated from a rice field in Shiga Prefecture, Japan (MAFF 236576, transferred from the National Institute of Agrobiological Science [NIAS], Japan).

DNA of the strain was extracted using NucleoBond AGX100 (TaKaRa Bio, Inc., Kusatsu, Shiga, Japan) after 9 days of cultivation at 25°C in 100 mL of potato dextrose broth (Difco). Extracted DNA was sheared to a size of ~20 kb using the g-TUBE (Covaris, Wobum, MA, USA). The DNA libraries were prepared from 10 µg of the sheared DNA with the SMRTBell template prep kit 1.0 reagents (Pacific Biosciences, Menlo Park, CA, USA) and size-selected according to the manufacturer’s protocol. The libraries were sequenced on a PacBio RS II instrument with the DNA sequencing reagent 4.0 and single-molecule real-time (SMRT) cells 8 Pac V3 (Pacific Biosciences). A total of 1.7 Gbp sequence data in 160,206 PacBio subreads were obtained from 2 cells of the SMRT cell. The prepared DNA was also sequenced using the Illumina MiSeq platform with the TruSeq DNA LT sample prep kit (Illumina, San Diego, CA, USA) to obtain 13.4-M paired-end (insertion length 557 bp, total 7.9 Gbp) short-read data, according to the manufacturers’ instructions.

The *de novo* genome assembly proceeded using the hybrid approach (5). First, Platanus (6) was used to perform De Bruijn graph assembly on the Illumina MiSeq reads and a total of 42.5Mbp highly accurate 34,389 fragments were retrieved. Next, DBG2OLC (7) was used to align these fragments onto the PacBio reads for consensus calling, resulting in a genome of 33.6 Mb in 139 contigs with an *N*_50_ value of 529,978 bp and an average G+C content of 51.3%. A total of 6,451 protein-coding genes were predicted using a combination of the gene prediction programs ALN (8) and GlimmerHMM (9).

The annotated genome sequence of *U. virens* IPU010 will contribute to the understanding of the pathogenicity of *U. virens*, which is important to the effort of securing reliable supplies of rice. Examining the genes responsible for biosynthesis of ustiloxins, which are the major mycotoxins produced by *U. virens* (10), is particularly important in terms of food safety. The ustiloxin biosynthetic gene cluster has recently been identified in the *U. virens* UV-8b genome (11, 12), and a homologous region to it is also found in the current genome sequence of IPU010. Analyses of genes co-expressed with the ustiloxin gene cluster will contribute greatly to controlling the toxicity of the fungus.

### Nucleotide sequence accession numbers.

This whole-genome shotgun project has been deposited in DDBJ/EMBL/GenBank under the accession no. BBTG00000000. The version described in this paper is the second version, BBTG02000000.
